# Assessment of serum high mobility group box 1 protein (HMGB1) levels and its change with treatment in patients with manic episode of bipolar disorder

**DOI:** 10.1192/j.eurpsy.2023.561

**Published:** 2023-07-19

**Authors:** A. Kara, Ö. D. Balaban, N. Karamustafalıoğlu

**Affiliations:** ^1^ Van Education and Research Hospital; ^2^Istanbul Bakirkoy Prof. Dr. Mazhar Osman Research and Training Hospital for Psychiatry, Neurology and Neurosurgery; ^3^Istanbul Bakirkoy Prof. Dr. Mazhar Osman Research and Training Hospital for Psychiatry, Neurology and Neurosurgery, İstanbul, Türkiye

## Abstract

**Introduction:**

There has been an increasing evidence in recent years that inflammation plays a role in the pathophysiology of bipolar disorder (BD). In addition to central inflammation, systemic immune response is thought to be associated with disease stages and course. HMGB1 protein is a member of damage-associated molecular pattern which plays a role in the regulation of cytokine release and is important for innate immunity. It has been revealed that HMGB1 levels change in many autoimmun and inflammatory diseases

**Objectives:**

Our study was designed to compare serum HMGB1 levels of inpatients with mania and healthy controls as well as analyzing its relationship with other inflammatory markers and disease severity. Another aim of our study is to determine the changes in serum HMGB1 levels before and after treatment in manic patients.

**Methods:**

Our study included 35 patients who were hospitalized in our hospital between November 2020 and April 2021, diagnosed with bipolar disorder, manic episode according to DSM-5 criteria, and 35 healthy controls who matched to the patient group in terms of age and gender. The sociodemographic and clinical characteristics data forms of the participants were filled in, and the patients were evaluated with the Young-Mania Rating Scale (YMRS) and the Hamilton Depression Scale (HAM-D). Serum HMGB1, CRP levels and complete blood count values were measured in healthy controls and patients before and after the treatment.

**Results:**

The main finding of our study is that HMGB1 levels did not show a statistically significant difference in the patient and healthy control groups (p>0.05). In addition, there was no significant change in serum HMGB1 levels of the patients after treatment compared to the level before treatment (p>0.05). Our study has also revealed that manic patients had a higher level of CRP than the ones in control group at a statistically significant level. The platelet/lymphocyte and neutrophil/lymphocyte rates of the patients increased after the treatment compared to the pre-treatment period (respectively p=0,026, p=0,003).

**Conclusions:**

The higher CRP levels in manic patients support the hypothesis of low-grade chronic inflammation, which is thought to be involved in the etiology of BD. The most important finding of this study is that there is no statistically significant difference of serum HMGB1 levels between the two groups. This can be explained by the fact that HMBG1 is one of the late mediators of inflammation and does not rise immediately during the acute period. However, since the inflammation process includes complex mechanisms involving many systems, it makes it difficult to comment on this issue.

**Disclosure of Interest:**

None Declared

